# Functional disruption of transferrin expression alters reproductive physiology in *Anopheles culicifacies*

**DOI:** 10.1371/journal.pone.0264523

**Published:** 2022-03-04

**Authors:** Jyoti Rani, Tanwee Das De, Charu Chauhan, Seena Kumari, Punita Sharma, Sanjay Tevatiya, Soumyananda Chakraborti, Kailash C. Pandey, Namita Singh, Rajnikant Dixit

**Affiliations:** 1 Laboratory of Host-Parasite Interaction Studies, ICMR-National Institute of Malaria Research, Dwarka, New Delhi, India; 2 Department of Biotechnology, Guru Jambheshwar University of Science & Technology, Hisar, India; Universidade Federal do Rio de Janeiro, BRAZIL

## Abstract

**Background:**

Iron metabolism is crucial to maintain optimal physiological homeostasis of every organism and any alteration of the iron concentration (i.e. deficit or excess) can have adverse consequences. Transferrins are glycoproteins that play important role in iron transportation and have been widely characterized in vertebrates and insects, but poorly studied in blood-feeding mosquitoes.

**Results:**

We characterized a 2102 bp long transcript *AcTrf1a* with complete CDS of 1872bp, and 226bp UTR region, encoding putative transferrin homolog protein from mosquito *An*. *culicifacies*. A detailed *in silico* analysis predicts *AcTrf1a* encodes 624 amino acid (aa) long polypeptide that carries transferrin domain. *AcTrf1a* also showed a putative N-linked glycosylation site, a characteristic feature of most of the mammalian transferrins and certain non-blood feeding insects. Structure modelling prediction confirms the presence of an iron-binding site at the N-terminal lobe of the transferrin. Our spatial and temporal expression analysis under altered pathophysiological conditions showed that *AcTrf1a* is abundantly expressed in the fat-body, ovary, and its response is significantly altered (enhanced) after blood meal uptake, and exogenous bacterial challenge. Additionally, non-heme iron supplementation of FeCl_3_ at 1 mM concentration not only augmented the *AcTrf1a* transcript expression in fat-body but also enhanced the reproductive fecundity of gravid adult female mosquitoes. RNAi-mediated knockdown of *AcTrf1a* causes a significant reduction in fecundity, confirming the important role of transferrin in oocyte maturation.

**Conclusion:**

All together our results advocate that detailed characterization of newly identified *AcTrf1a* transcript may help to select it as a unique target to impair the mosquito reproductive outcome.

## Introduction

Iron is a trace biologically indispensable and potentially toxic element. All living organisms require iron for innumerable biological functions such as energy generation *via* electron transport, oxygen transport, DNA synthesis and repair, immunity, healing, melanization, and as a biocatalyst for various processes [1–3]. In addition, in anautogenous insects, blood meal iron is an essential requirement for ovarian follicles growth and oocyte development [4, 5]. A surplus amount of iron leads to hydroxyl radical production which may cause cell/tissue damage, and impact the overall performance of an individual. Therefore, maintaining an optimal level of iron is crucial for the survival of every biological organism [6–9]. Several studies on the vertebrate system highlight that transferrin is a major iron transport carrier in the blood [10, 11]. Among the four well-characterized mammalian transferrins (serum transferrin, lactotransferrin, melanotransferrin, and inhibitor of carbonic anhydrase) only transferrin and lactotransferrin are secretary in nature, and binds to iron with a greater affinity [12]. Although, transferrin superfamily members have been reported in more than 34 invertebrate species, including insects, very little is known about its role in iron transportation in mosquito species [13, 14]. Among all those proteins involved in iron transport, Transferrin and Ferritin gained the most attention, because of their unique structure and their unique iron-binding ability [1].

Insect transferrins, having a molecular weight of ~ 66 kDa, are identified as juvenile hormone-regulated proteins, and function as potent immune proteins, antibiotic agents, antioxidants, vitellogenin proteins and protect from plant secondary metabolites, etc. [15, 16]. *Kurama et*. *al*., have suggested that transferrin is involved in vitellogenesis in flesh flies (*Sarcophaga peregrina*), utilized by developing oocytes [17, 18]. Besides these roles, transferrin protein is also found as the utmost component of milk composition in tsetse flies for intrauterine larval development [19]. However, unlike vertebrate transferrin, insect transferrin evolved with only an N- terminal iron-binding pocket for iron-binding and transportation [20, 21]. Although this strategy, seems to sacrifice half of the iron-binding capacity of the transferrin to defeat the “piracy” action of the pathogen, but the molecular basis of iron transportation is still sparse [22].

Anautogenous mosquitoes take an iron-rich blood meal for their egg maturation and completion of the gonotrophic cycle [23–25]. Rapid utilization of this heavy blood meal iron content may also result in the formation of hydroxyl free radicals that are highly destructive [26, 27]. To minimize this oxidative effect insects are evolved with a unique ability to mobilize, utilize and store iron through various iron-binding proteins, such as ferritin (storage) and transferrin (transportation) [8]. Insect homolog transferrin has also been identified from several mosquito species, and their immune role has been explored in a few mosquitoes such as *Ae*. *aegypti* [28, 29], *An*. *gambiae*, and *Cu*. *quinquefasciatus* [30]. For e.g., an *in-vitro* study on *Cu*. *pipiens pallens*, showed prolific expression in cypermethrin-resistance strain, advocating that transferrin may confer insecticide resistance against cypermethrin [31]. However, so far functional correlation of transferrin in the reproductive physiology of any mosquito species remains unexplored. Literature suggests that many insects (including *D*. *melanogaster*, *Ae*. *aegypti*, and *An*. *gambiae*) genome also encodes transferrin 2, 3, and 4 in addition to transferrin1 [15].

*An*. *culicifacies*, is a major vector responsible for 65% of malarial cases in rural India [32]. Control of *An*. *culicifacies* is very challenging because of the development of insecticide resistance, and the biology of this mosquito remains poorly understood. In the present study, we describe *An*. *culicifacies* transferrin 1 (*AcTrf1a)* which shares the highest 80% identity with *An*. *stephensi* followed by 74%, 52%, and 21% identity with *An*. *gambiae*, *Ae*. *aegypti* and *Ho*. *sapiens* transferrin, respectively (S6 Table in S1 File). We also reported the differential expression of *AcTrf1a* transcript in response to the development, blood meal, iron, and bacterial challenge. Finally, using the functional genomics approach we showed that *AcTrf1a* play important role in shaping the reproductive fecundity of mosquito *An*. *culicifacies*.

## Material methods

### *In silico* analysis: Domain arrangement and structural modelling of *AcTrf1a*

The putative *AcTrf1a* gene was retrieved from the hemocyte RNA-Seq data of naïve *An*. *culicifacies*. Initial BLASTx analysis against the NCBI NR database showed a significant hit to the transferrin-like proteins originating from multiple mosquitoes and insect species. To retrieve a full-length transcript, BLASTn analysis was performed against the genome predicted transcript database of the *An*. *culicifacies* mosquito, which is available on *www*.*vectorbase*.*org*. Top 10–15 blast hits FASTA sequences were selected from mosquito and non-mosquito species, followed by alignment using ClustalX2 for multiple sequence alignment analysis. The phylogenetic tree was generated by providing an aligned file of ClustalX2 as an input in the maximal likelihood program of MEGAX software. The program was run on 1000 bootstraps to confers branching reliability [33]. *AcTrf1a* domain annotation was performed using online web server Pfam (http://pfam.xfam.org/). The iron-binding site of *AcTrf1a* was determined using sequence alignment with human transferrin (GenBank ID: NP_001054.1). CASTp (http://sts.bioe.uic.edu/castp/index.html?1bxw) was used for protein cavity analysis. Almost all proteins carries cavity and pockets in their structure for various purposes including metal and ligand binding, for transferrin we analysed cavity in order to determine detail microenviroment of iron binding site and its surrounding. Homology modelling of *AcTrf1a* (sequence and other details of *AcTrf1a* are provided in S1 and S2 Tables in S1 File) was performed using three independent automated online servers Phyre2 (http://www.sbg.bio.ic.ac.uk/~phyre2/html/page.cgi?id=index), RaptorX (http://raptorx.uchicago.edu/) and Robetta (https://robetta.bakerlab.org/). The quality of different protein models developed by different servers was assessed by SAVES v6.0 (https://saves.mbi.ucla.edu/), and Robetta generated model was found the best and selected for further analysis. Structural visualization of protein was carried out using Pymol (https://pymol.org/2/). N-linked glycosylation site prediction was done by Gene Runner software on default parameters and further validated by CBS prediction servers (http://www.cbs.dtu.dk/services/TargetP/).

### Mosquito rearing

*An*. *culicifacies* (sibling species A) was reared and maintained in the central insectary of the National Institute of Malaria Research under standard rearing condition of 28 ± 2°C, relative humidity 60–80%, and 12:12 hr light/dark cycle. Aquatic stages were reared in enamel trays with water supplemented with a mixture of dog food and fish food. After emergence adults were kept in cages and fed on cotton swabs dipped in 10% sugar solution. The ethical committee of NIMR has approved all the steps taken for rearing and maintaining a cyclic colony of mosquitoes.

### Bacterial challenge assays

Exogenous bacterial challenge was posed by injecting bacterial culture of *Escherichia coli* and *Bacillus subtilis* in 0–2 days old female mosquitoes using nanoinjector facility (Drummond Scientific Nanoject II, Broomall, PA, USA). Both *E*. *coli* and *B*. *subtilis* were cultured in Luria Bertani broth as the overnight culture at 37°C. Bacterial cultures at the log phase were centrifuged to 2000g for 10min. After washing pellet 2–3 times with phosphate-buffered saline (PBS), were resuspended in PBS buffer. For heat-killed assays bacterial cells dissolved in PBS having OD-0.91 and 0.96 for *E*. *coli* and *B*. *subtilis*, respectively, were kept at 90°-120°C for 10 min to expose pathogen-associated molecular patterns (PAMPs). Mosquitoes after the challenge with the heat-killed bacterial soup were placed in plastic cups, having moistened surface with wet filter paper-pad. Regular water and sugar supply through wet cotton swabs and raisins were maintained on the net for better recovery under standard rearing conditions. The experimental design consisted of two different treatment groups: the ‘bacterial challenge’ group was injected with 69 nl of bacterial solution and the control group with 69 nl of PBS buffer from the same cohort of mosquitoes. Post injection, around 30–35 mosquitoes were dissected for hemocyte tissue collection in a time-dependent manner. Each group and time point were replicated three times.

### Exogenous ferric chloride feeding assay

For non-heme iron feeding, post emergence mosquitoes were either fed on 10% sucrose solution alone (control) or sugar supplemented with FeCl_3_ till the end of the experiment. Both sugar and supplemented meal were replaced with a fresh meal every 24 hr. After 2–3 days of supplementation, fat-body tissue was collected from 20–25 mosquitoes from both the control and iron supplemented group to check the transcriptional response of selected transcripts.

### *dsRNA* mediated gene knockdown

For *AcTrf1a* knockdown, initially, we amplified the single-stranded complementary DNA by applying PCR amplification strategy using dsRNA primers carrying T7 overhang (S3 Table in S1 File). The purified PCR product was quantified (Nanodrop 2000 spectrophotometer, Thermo scientific), and validated (agarose gel electrophoresis) using Thermo Scientific Gene JET PCR Purification Kit (Cat #K0701). Next, purified PCR product was subjected to double-stranded RNA synthesis using Transcript Aid T7 high-yield transcription kit (Cat# K044, Ambion, USA). After purification, ~69 nl (~3ug/ul) of purified dsRNA product was injected into the thorax of newly emerged (1–2 day old) and cold anesthetized mosquitoes using a nano-injector facility (Drummond Scientific, CA, USA). An equal number of age-matched mosquitoes were also injected with *dsLacZ* (bacterial origin) as a control group. 3-4-days post-injection desired samples such as fat-body and ovary were collected from 20–25 mosquitoes and examined for silencing efficiency by quantitative PCR.

### Mosquito tissue sample collection

Before tissue collection, adult mosquitoes were anesthetized by putting them at 4°C for 4–5 min. Later, placed on to dissecting slide under the microscope and various tissue like fat-body, hemocytes, midgut, salivary gland, ovary, spermatheca, and male reproductive organs were collected as described earlier [34]. For hemolymph collection, approx. 2–3 μl of anticoagulant consisting of 60% Schneider’s medium, 10% fetal bovine serum, and 30% citrate buffer is injected into the thorax. Mosquito belly bulged out, and then a small incision was made in the abdomen using a microscopic needle so that transparent hemolymph oozes out. Hemolymph was collected using a micropipette and pooled in Trizol. Fat- body was collected by pulling all the abdominal tissues from the last two abdominal segments, and afterward, force tapping of the abdominal carcass was done so that pale yellow color fat-body oozes. Developmental stages (egg, four larval instars, and pupa) were collected independently in Trizol.

### Total RNA isolation and cDNA synthesis

Trizol and alcohol extraction method was used for isolation of total RNA manually from pooled samples, as previously described [35]. After quantification by Nanodrop 2000 spectrophotometer (Thermo scientific), 1 μg of total RNA extracted was subjected for the synthesis of the first strand of cDNA using verso cDNA synthesis kit (Thermo Scientific, USA). Actin was used as a reference gene for quality validation of sscDNA.

### Gene expression analysis

Differential expression of selected transcripts was done using RT-PCR. Primers were designed (https://primer3plus.com/) using either sequenced cDNA or vector base extracted sequences (S3 Table in S1 File). SYBR green qPCR master mix and Bio-Rad real-time machine was used for relative expression analysis of selected targets under different biological conditions. qPCR protocols remained the same for all sets of primers with an initial denaturation at 95°C for 5 min, 40 cycles of 10 sec at 95° C, 15 sec at 52° C, and 22 sec at 72°C. At the end of each cycle, fluorescence reading was recorded at 72°C. In the final steps, PCR at 95° C for 15 sec followed by 55° C for 15 sec and again at 95° C for 15 sec were completed before generating a melting curve. Three independent biological replicates for each experiment were run using the above protocol. The actin gene was used as an internal control (housekeeping genes) and the relative quantification data were analysed by the 2^-ΔΔCt^ method [36]. And final relative expression graphs were plotted using GraphPad Prism7.04 and the significance of treatment was determined by multiple comparison using one-way ANOVA.

### Assessment of ovary development

All experimental groups including control, iron supplemented, and silenced were offered blood meal on the rabbit. Only fully fed mosquitoes were selected for further experimentation, while partial fed or unfed mosquitoes were discarded. About 72 hr post blood meal, mosquitoes were anesthetized and placed over dissecting slides under a binocular microscope for ovary development assessment. Ovaries were dissected in PBS and the number of oocytes developed inside the matured ovary was counted manually and compared with control mosquitoes.

### Neutral red staining

Mosquitoes from both control and *AcTrf1a* silenced regime were blood-fed as previously described. Mosquitoes were anesthetized prior to dissection by chilling at 4° C for 3–5 min. At 72 hr of blood meal, ovaries were dissected in 1x PBS and stained with 0.5% neutral red solution in acetate buffer (Sigma–Aldrich, St. Louis, MO). After staining, ovaries were again rinsed in PBS buffer and placed under a coverslip. Later these ovaries were visualized under a microscope for previtellogenic imaging of the ovary.

### Statistical analysis

For statistical analysis, test sample data were compared with the control data set using GraphPad Prism 7.04 software, and treatment differences were determined one-way ANOVA, using turkey’s multiple comparison. The result of individual replicates were plotted as scatter plot with bar at mean and SD value. The *Mann–Whitney U test* was used to evaluate and analyse the number of developing follicles inside the ovary of gravid female mosquitoes. A final *p*-value of less than 0.05 level was considered significant for both tests. All experiments were conducted thrice for data validation.

## Results

### 1. Molecular characterization of *AcTrf1a*

**Fig 1:**
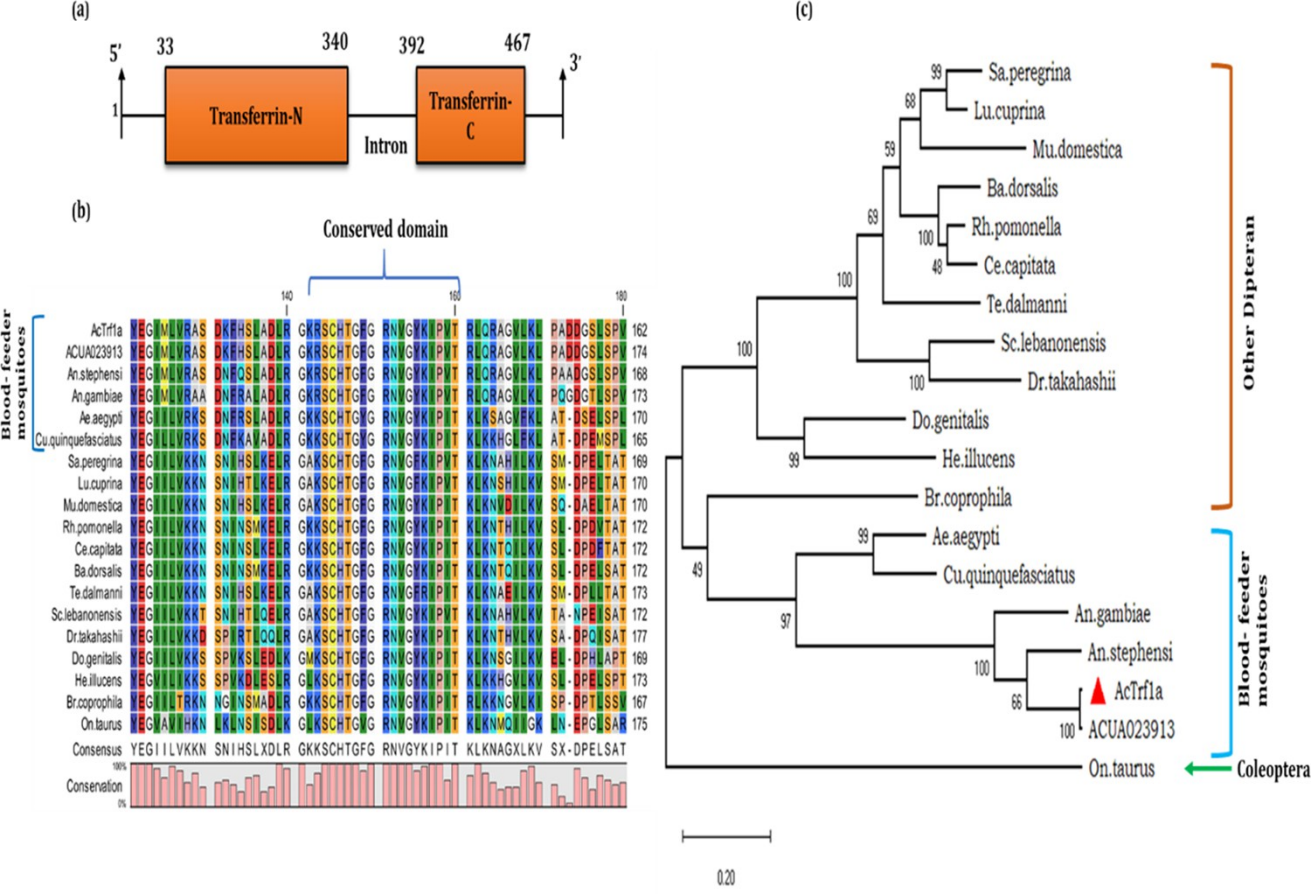
Sequence analysis of *An*. *culicifacies* hemocytes transcript encoding transferrin1 protein: (a) Genomic organization and domains annotation of *An*. *culicifacies* transferrin1 (*AcTrf1a)*; (b) The multiple sequence alignment snapshot of highly conserved TRF domain region, capture from full-length sequence aligment of the selected insect transferrin homolog proteins (S6 Fig in S1 File); (c) Phylogenetic analysis of the selected transcripts was done using maximum likelihood algorithm upon 1000 times bootstraps in MEGAX software. The percentage of trees in which the associated taxa clustered together is shown next to the branches. The tree is drawn to scale, with branch lengths measured in the number of substitutions per site. *Onthophagus taurus* (Coleoptera) transferrin sequence (720 aa) was taken as an outgroup for analysis. *AcTrf1a*, an allelic form of genome predicted full-length transcript ACUA023913-RA, of the mosquito *An*. *culicifacies*; *An*. *gambiae* (XP_310734.4); *An*. *stephensi* (XP_035908482.1); *Ae*. *aegypti* (AAB87414.1); *Cu*. *quinquefasciatus* (EDS42355.1); *Sa*. *peregrina* (Q26643.1); *Lu*. *cuprina* (XP_023295921.1); *Mu*. *domestica* (ADU25046.1), *Rh*. *pomonella* (XP_036318323.1); *Ce*. *capitate* (XP_004524870.1; *Ba*. *dorsalis* (AIA24538.1); *Te*. *dalmanni* (XP_037937085.1); *Sc*. *lebanonensis* (XP_030379051.1); *Do*. *genitalis* (AYV99626.1), *He*. *illucens* (XP_037919193.1); *Br*. *coprophila* (XP_037051978.1); *Dr*. *takahashii* (XP_017002431.1); *On*. *taurus* (XP_022906419.1).

While analysing RNA-Seq data of the hemocyte, we identified two transcripts, encoding putative transferrin homolog proteins, from the mosquito *An*. *culicifacies* [37]. An initial BLASTx homology search against a non-redundant database indicated that both transcripts encode transferrin domain (Fig 1A). In this analysis, we observed that only one transcript (2102bp) carries the highest homology to insects (transferrin1) but not the other (2162 bp) which showed unusual clustering with vertebrates’ transferrin proteins (S1 Fig in S1 File). Here, we analysed the nature and function of the insect’s transferrin homolog transcript in detail. BLASTn analysis against draft genome database of the mosquito *An*. *culicifacies*, showed 99% identity to the predicted transcript (ACUA023913-RA), with at least four single nucleotide substitutions (S4 Table in S1 File). This observation suggested the hemocyte originated transcript is an allelic variant of genome predicted (ACUA023913-RA) transcript, with slight modification of amino acid (aa) sequences (S4 Table in S1 File). A comparative physiochemical properties analysis with genome predicted full-length transcript (ACUA023913-RA), indicated that identified *AcTrf1a* is partial, and lacks signal peptide sequence (S2 Table in S1 File). A full-length multiple-sequence alignment of *AcTrf1a* encoded 624 AA long peptide with its homologs from diverse a class of insects showed a high degree of sequence conservation, among blood feeder mosquitoes, (S1 Fig in S1 File.; Fig 1B). The phylogenetic tree obtained at maximum likelihood is shown (Fig 1C). We observed a close relatedness of *AcTrf1a* among mosquito species, by forming a separate cluster with mosquito transferrin homologs, then other insect clades.

**Fig 2:**
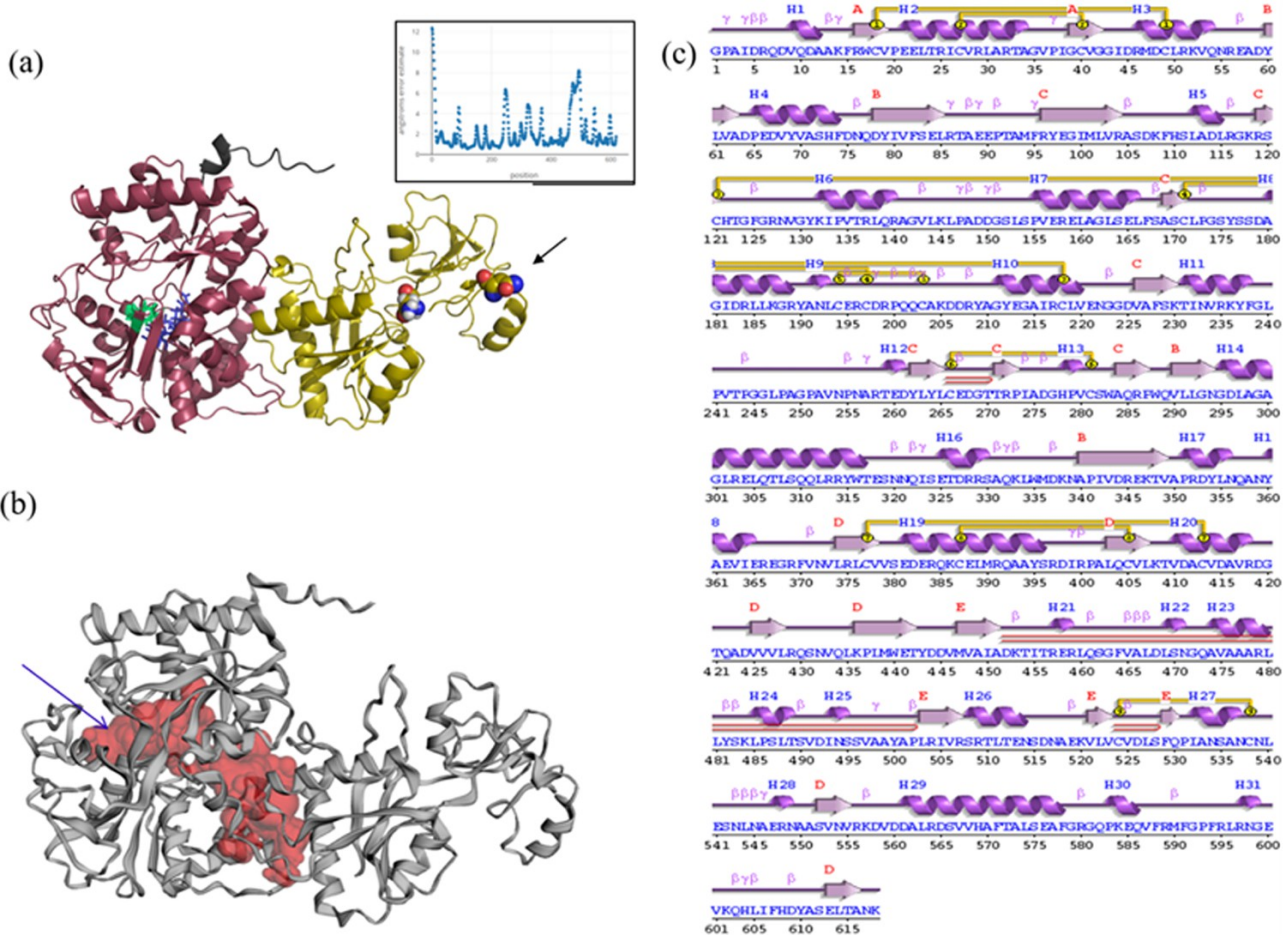
Domain analysis and structural modeling of *AcTrf1a*. (a) Cartoon representation of *AcTrf1a*, protein model was generated using web-based protein modelling server Robetta (https://robetta.bakerlab.org). In the cartoon, the N-terminal domain (N-lobe) of transferrin is shown in dark red, while iron and anion binding residues in green and blue respectively, inset graph is showing the reliability of the protein model across its sequence directly obtained from Robetta (https://robetta.bakerlab.org) server. Glycosylation modification sites were represented in multi-color sphere (asparagine residues) and interestingly both the glycosylation sites were found in the C-terminal lobe of *AcTrf1a*, arrow indicates site with superior glycosylation activity. pymol (http://www.pymol.org) was used for visualization purpose; (b) Cavity analysis of *AcTrf1a* was carried out using Castp server (http://sts.bioe.uic.edu/castp/index.html?2cpk), the biggest pocket present in N-lobe encompasses iron-binding site; (c) The output from PDBsum (http://www.ebi.ac.uk/thornton-srv/databases/cgi-bin/pdbsum/GetPage.pl?pdbcode=index.html) run on the sequence of *AcTrf1a* shows secondary structural elements present in the modelled protein along with locations of cystine residues.

Our domain prediction and annotation analysis indicated that *AcTrf1a* (Fig 1A), encodes a functional N-terminal lobe (domain), capable of iron-binding. Whereas the C-terminal domain is partial/ truncated and most of the known (conserved) iron-binding residues are missing in this domain (S5 Table in S1 File). Structural modelling further verifies that the N-terminal transferrin domain carries a regular lobe-like structure (Fig 2A; S5 Table in S1 File) along with cavity and conserved residues necessary for iron-binding. We also noticed that the C-terminal domain is much more elongated i.e. less compact, than the N-terminal domain, and lack iron-binding site residues and cavity (Fig 2B and S5 Table in S1 File). However, bioinformatic software-based prediction of two putative N-glycosylation sites at asparagine (Asn, N) amino acid residue in the C-terminal lobe of transferrin1 at 493 and 500 position of amino acid. (Fig 2B and S4 Table in S1 File), indicated that this newly identified *AcTrf1a* is an allelic variant of the ACUA023913-RA transcript. The secondary structure arrangement of the full protein sequence is shown in Fig 2C. The modelled *AcTrf1a* structure is composed of 31 helices, and a total of 18 cystine residues all are predicted to be linked via disulphide bonds.

### 2. Developmental and tissue-specific transcriptional response of *AcTrf1a*

**Fig 3:**
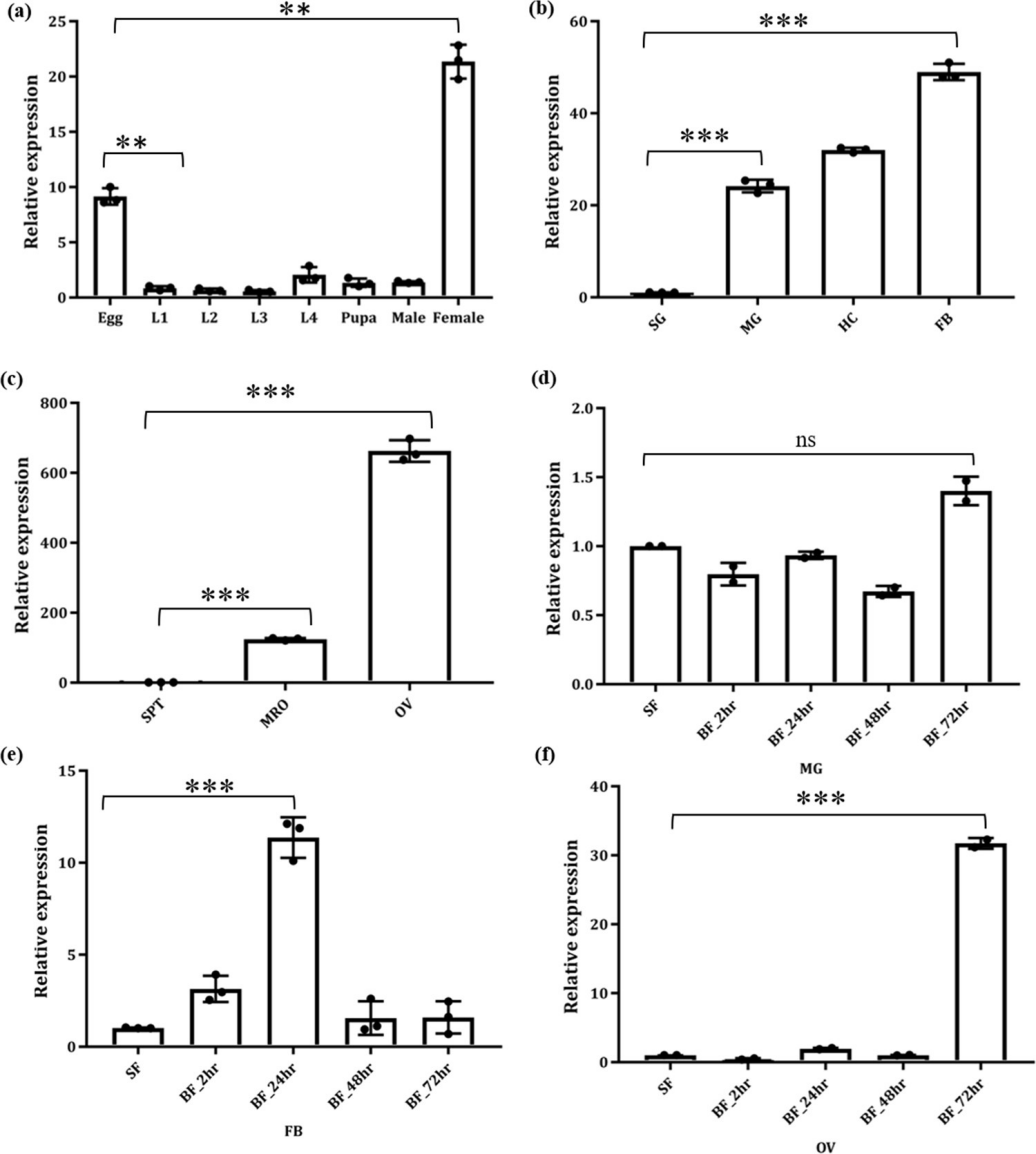
Spatial-Temporal expression profiling of *AcTrf1a* in the mosquito *An*. *culicifacies* (a) Relative gene expression during mosquito development, showing an abundance of *AcTrf1a* transcript in the eggs *(p<0*.*0018)* and adult female mosquito *(p<0*.*0045)*. The first instar larval stage was considered as a control for all test samples. L1-4 (larval stages) (n = 10, N = 3); (b) Expression analysis in the adult female mosquito tissues showing multi-fold upregulation of *AcTrf1a* in fat-body (FB/*p<0*.*0001*), hemocytes (HC/*p<0*.*0001*) and midgut (MG/*p<0*.*0019*) respectively. Data were analysed relative to salivary gland (SG) (n = 25, N = 3); (c) *AcTrf1a* mRNA levels profiling in the mosquito reproductive tissues shows a prolific expression in the ovary (OV/*p<0*.*0003*) and male reproductive tissue (MRO/*p<0*.*0001*). Female spermatheca was set as a control for relative analysis (n = 20, N = 3); (d) Tissue-specific transcriptional response of *AcTrf1a* after blood-feeding: mosquito tissues such as midgut, fat-body, and ovary were collected at 2, 24, 48, and 72 hr post blood meal (PBM) and compared with sugar-fed naïve female as a control (n = 25, N3). *AcTrf1a* mRNA is minimally expressed in the blood-fed midgut (ns); (e) moderate in fat-body (*p<0*.*0003*) post 24 hr of blood meal, and (f) highest in 72 hr PBM ovary (*p<0*.*0005*). All the three independent biological replicates are shown using dots and statistical analysis was performed by one-way ANOVA where asterisks represented *p < 0.05; **p < 0.005; and ***p < 0.0005, (*n* = represents the number of mosquitoes pooled for sample collection; *N* = number of replicates).

While we observed an abundant expression of *AcTrf1a* in the eggs and whole adult female body (Fig 3A), a tissue-specific transcriptional profiling showed a dominant expression in fat-body and hemocyte (Fig 3B), of the 3–4 days old naive adult female mosquitoes. Interestingly, we also noticed relatively higher expression of *AcTrf1a* in the female ovary than the male reproductive organ (Fig 3C). These data suggested that co-expression of *AcTrf1a* in the fat-body and reproductive organs of the naïve adult female mosquitoes may likely have an important role in mosquito reproduction and egg development.

### 3. Spatio-temporal expression pattern of *AcTrf1a* following blood-feeding

Host blood meals serve as a rich source of resources for vitellogenesis and follicles maturation in adult female mosquitoes. To explore the possible effect of blood meal, a time-dependent transcriptional response of *AcTrf1a* was monitored in the target tissues such as midgut, fat-body, and ovary, *‘engaged in*’ digestion and nutrient uptake. 3-4-days old adult female mosquitoes were fed on rabbits, and fully engorged females were dissected for tissues collection at a desired time after a blood meal. Compared to the midgut (Fig 3D), a significant modulation in the expression of *AcTrf1a* was observed in the fat-body and ovary. A gradual enrichment in the *AcTrf1a* transcript was observed in fat-body till 24 hr post blood meal (Fig 3E), which was restored to basal level within 48 hr of blood-feeding. However, in the ovary we observed heightened expression level of *AcTrf1a* post 72 hr of blood meal (Fig 3F). Altogether, these data correlate a bi-phasic regulation of transferrin expression of *AcTrf1a* in the fat-body and ovary, possibly to meet the requirement of iron during ovary maturation and egg development, though further investigation is needed.

### 4. Exogenous bacterial challenge differentially regulates *AcTrf1a*

**Fig 4:**
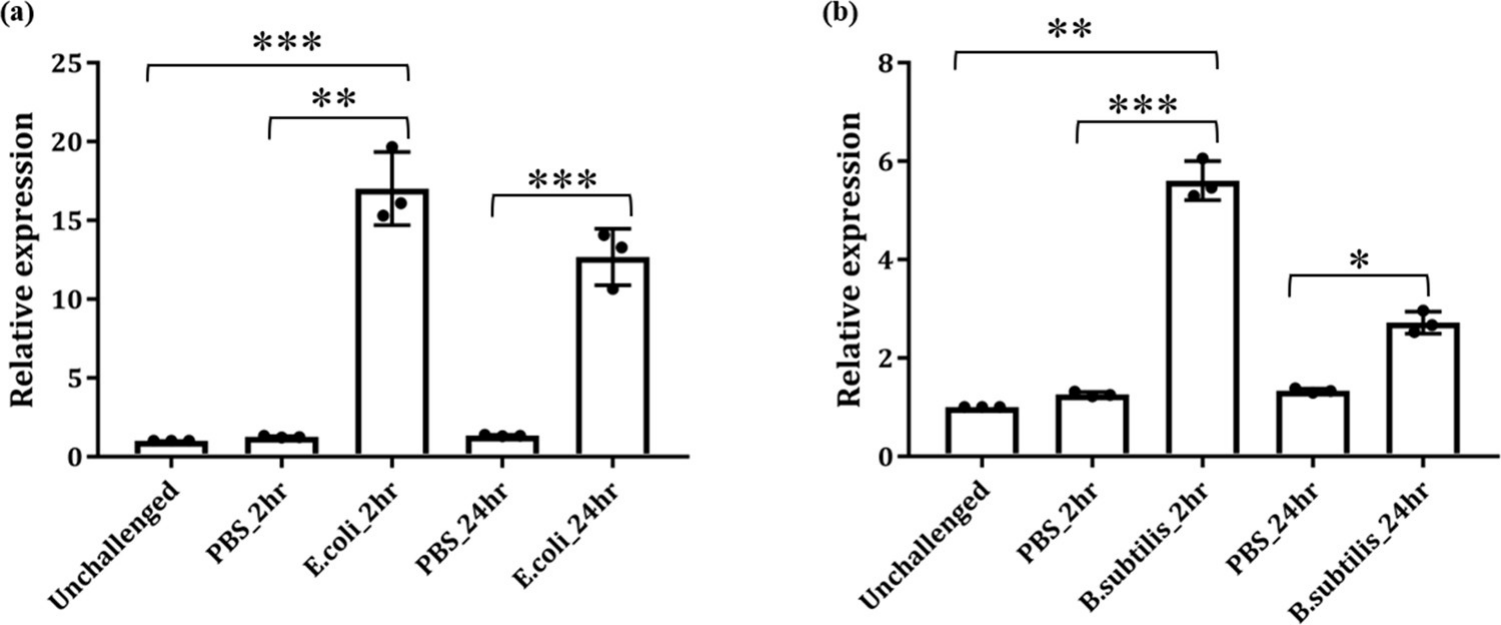
Transcriptional response of *AcTrf1a* against bacterial challenge. 3–4 days old adult female mosquitoes were injected either with 69 nl of heat-killed bacterial suspension in PBS or PBS alone as a control in mosquito thorax (n = 30–35, N3). After recovery, time-dependent series, hemocyte samples were collected post 2 and 24, hrs of bacterial challenge. (a) Relative transcriptional profiling against *E*. *coli* and; (b) *Bacillus subtilis* showing the immune responsiveness of *AcTrf1a* to bacterial challenge. Data represented in the figure were from three independent biological replicates, where each dot is equivalent to replicate. Statistical analysis was done using one way ANOVA *viz* *p < 0.05; **p < 0.005; and ***p < 0.0005, (*n* = represents the number of mosquitoes pooled for sample collection; *N* = number of replicates).

Several studies highlight that insect transferrin serves as an acute-phase protein and play important anti-bacterial immune role [15, 22, 38]. Therefore, we also tested whether *AcTrf1a* expression is modulated in response to exogenous bacterial challenge in the naïve mosquito. We injected 2–3 days old adult female mosquito of *An*. *culicifacies* with heat-killed bacterial suspension of *E*. *coli* and *B*. *subtilis* (OD_600 =_ 0.9–1). Injections of saline water were considered as a control for baseline expression. Post injection, hemocytes were collected from 30–35 mosquitoes at a different time intervals. We observed that irrespective of the nature of injected bacteria gram-negative or gram-positive (Fig 4A and 4B), *AcTrf1a* transcript expression was highly upregulated within two hrs of the bacterial challenge. Together, these observations further strengthen the hypothesis that *AcTrf1a* has an antibacterial role in mosquito *An*. *culicifacies*.

### 5. Effect of the iron supplement on *AcTrf1a* expression and oocyte load

**Fig 5:**
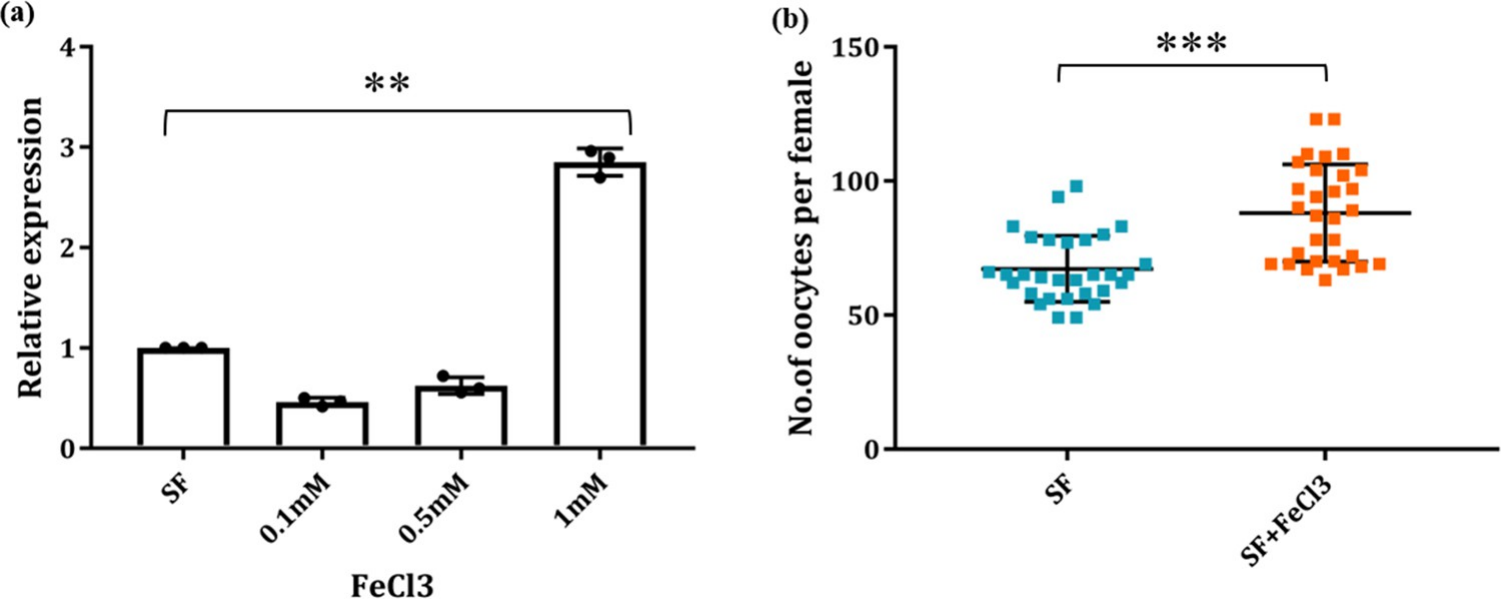
mRNA level augmentation of *AcTrf1a* transcripts on iron supplemented diet and effect on the mosquito fecundity. Female mosquitoes were allowed to feed on different iron concentrations prepared in sugar solution and the mRNA level of target transcripts was measured in the fat-body. (a) Comparative transcriptional profiling of *AcTrf1a* in fat-body tissue collected from control and treatment groups. An enriched expression (*p<0*.*0018*) was observed at 1 mM conc. of iron supplemented diet (n = 20–25, N = 3); Data represented in the figure were from three independent biological replicates, where each dot is equivalent to replicate. Statistical analysis was done using one-way ANOVA *viz* *p < 0.05; **p < 0.005; and ***p < 0.0005, (*n* = represents the number of mosquitoes pooled for sample collection; *N* = number of replicates); (b) Dot plot showing that iron supplemented diet during the early stage of development just after eclosion leads to enrichment of oocytes (*p<0*.*00001*) maturing inside the female ovary. Both the control (SF) and test group (SF+ FeCl_3_) were allowed to feed on rabbit blood and subsequently kept for ovary assessment for oocyte count (n = 10, N = 3). Data represented in the figure were from three independent biological replicates, where each dot represents number of oocytes present in mature ovary of female mosquito. Statistical analysis was done using *Mann–Whitney U test* (*n* = represents the number of mosquitoes pooled for sample collection; *N* = number of replicates).

Next, to test the possible role of *AcTrf1a* in reproductive physiology, first, we evaluated whether supplementation of non-heme iron modulates the mosquito transferrin’s expression. To ensure an optimal impact, initially, we offered two independent regimes of different serial dilution of ferric chloride i.e., 1 μM, 5 μM, 10 μM, and 0.1 mM, 0.5 Mm, 1 mM in 10% sugar solution female mosquito just after emergence *via* oral feeding. After 48–72 hr of iron feeding, fat-body and midgut tissues were collected, and the transcription profile of *AcTrf1a* was monitored through Real-Time PCR assay. Compared to low concentration, which showed a mild impact (S2 Fig in S1 File), significant upregulation was noticed only in fat body not in the midgut of naïve mosquitoes (S4 Fig in S1 File), when fed with a concentration of 1 mM FeCl_3_ (Fig 5A).

With this optimization, next we investigated whether this extra iron supplementation (1 mM FeCl_3_) influences mosquito reproductive physiology such as the number of developing oocytes inside the ovary. To perform this assay, mosquitoes were kept under two nutritional status: 1) 10% sugar solution only or 2) 10% sugar supplemented with ferric chloride solution (1 mM). Post supplementation, both groups of mosquitoes were offered rabbit blood for successful completion of the first gonotrophic cycle. After 72 hr of blood meal, ten mosquitoes from each control and test group were dissected and examined for ovary development. Mosquitoes fed with 10% sugar alone had an average of 60 mature oocytes per female. While mosquitoes kept on iron supplemented diet, yielded an average of 75 oocytes/female mosquitoes (Fig 5B). This data highlighted that *AcTrf1a* may have an important role in the mosquito reproductive physiology, possibly by altering previtellogenic nutritional iron transport and status of the resting stage.

### 6. Transferrin knockdown reduces oocyte count in the ovary

**Fig 6:**
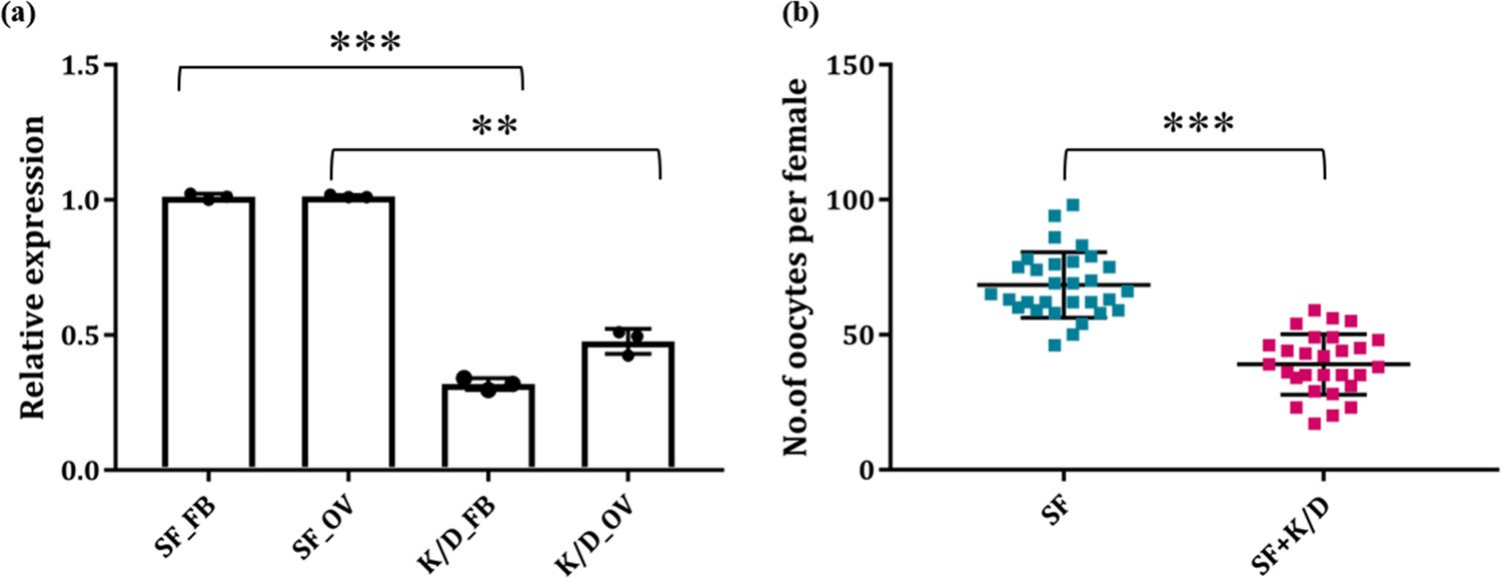
*AcTrf1a* silencing effect on mosquito oocyte development. (a) Real time-PCR based knockdown validation of *AcTrf1a* in fat-body (*p<0*.*0001*) and ovary (*p<0*.*0052*) tissue after *dsAcTrf1a* injection to newly emerged mosquitoes in comparison to same age group *dsLacZ* injected (n = 25, N = 3). Data represented in the figure were from three independent biological replicates, where each dot is equivalent to replicate. Statistical analysis was done using one-way ANOVA *viz* *p < 0.05; **p < 0.005; and ***p < 0.0005, (*n* = represents the number of mosquitoes pooled for sample collection; *N* = number of replicates); FB, fat-body; OV, ovary. (b) Dot plot showing the comparative oocyte count reduction (*p<0*.*00001*) post *AcTrf1a* knockdown in female ovary tissue compared to control mosquito group. Post dsr injection mosquitoes were fed on blood meal and ovary assessment was done, (n = 10, *N* = 3). Data represented in the figure were from three independent biological replicates, where each dot represent number of mature follicles per female. Statistical analysis was done using *Mann–Whitney U test* (*n* = represents the number of mosquitoes pooled for sample collection; *N* = number of replicates).

To further evaluate and support the above hypothesis, we performed a dsNA mediated gene knockdown/silencing experiment. For this, 2-day old mosquitoes were injected with double-stranded RNA of *AcTrf1a*, and mosquitoes were kept under optimal insectary conditions for better recovery. Fat-body and ovary tissues were collected from 20–25 mosquitoes three-day post silencing to check the transcript mRNA level. Compared to the *dsLacZ* injected control mosquito group, we observed at least a 70% reduction in the transcript of *AcTrf1a* expression in the fat-body and 50% in the ovary of knockdown mosquitoes (Fig 6A). Both control and the silenced group were offered blood meal and after 72hr of blood meal, individual mosquito (n = 10) was dissected in PBS and a total number of mature oocytes were manually counted under a microscope. Our initial phase-contrast microscopic analysis showed a significant difference in maturing follicles after silencing (S3 Fig in S1 File). We observed that in contrast to control mosquito which has 63 (average) oocyte per mosquito, silenced mosquitoes showed only 40 (average) oocytes per mosquito (Fig 6B). Additional supplementation of non-heme iron to silenced mosquitoes did not altered the oocyte load (S5 Fig in S1 File), confirming that *AcTrf1a* has a direct influence on mosquito’s reproductive physiology.

## Discussion

Transferrin family is a large group of protein found in both vertebrates as well as invertebrates, including insects, and play an important role in iron transportation and metabolism [39–42]. The majority of family members have two lobes for iron-binding which evolved *via* gene duplication events during evolution [12, 43]. Transferrin protein is pleiotropic in insects, involved in iron homeostasis, immunity, vitellogenin protein, antibacterial agent, and sequestered uptake in ovarian follicles [15, 17, 44–46].

Although, several studies highlight the antibacterial role of transferrin in the adult female mosquitoes, that feed on iron-rich blood, its role in ovarian development and egg maturation, remains poorly understood. Here, we have identified and characterized an iron-binding transferrin allelic variant *(AcTrf1a)* from the hemocyte transcriptome of Indian malarial vector *An*. *culicifacies*. Through comprehensive spatial/temporal expressional profiling, and functional knockout studies, for the first time we demonstrate that mosquito transferrin significantly influences the reproductive physiology of the mosquito *An*. *culicifacies*. The initial finding of transferrin transcripts from the hemocyte transcriptome corroborates with the previous observation that the transferrin is expressed dominantly in the fat-body, and hemolymph [13, 16]. Unlike, vertebrates and insects, the blood-feeding mosquito transferrin lacks an N-Glycosylation site [15]. But the prediction of N-Glycosylation site in the hemocyte originated transferrin (*AcTrf1a)*, indicates that this is an allelic variant of ACUA023913-RA (genome predicted transcript), which shares 99.36% identity at amino-acid level (S6 Table in S1 File). Recent structural studies of insect transferrin show that the iron-binding mechanism of insect transferrin (*Trf1*) is completely different compared to another known vertebrate transferrin. Insects transferrin (*Manduca sexta*) iron-binding site is localized at N-terminus and coordinated by two tyrosine ligands, and two CO_3_^2-^ anions, contrary to vertebrate transferrin where both N- and C-lobe participate in iron-binding [47]. Furthermore, in vertebrate transferrin iron-binding site is constituted of two tyrosines, one aspartate, one histidine, and one carbonate anion [48, 49]. A dominant expression of *AcTrf1a* in the egg than other developmental stages such as larva, pupae, and in 3–4 days old adult female than age-matched male mosquitoes, together indicates that *AcTrf1a* may play a key role in the egg development of this mosquito species. A similar expression pattern for other iron transport proteins i.e., ferritin has also been observed in *Aedes* [50]. Compared to fat-body/hemocyte, an observation of a multifold enriched expression in the reproductive organs of both sexes, further strengthen the hypothesis that *AcTrf1a* may likely have a potential role in the transportation, and optimal iron supply maintenance in the sexually maturing reproductive organs.

In hematophagous insects, intake of iron-rich blood meal is crucial for oocyte development, ovary maturation, and successful completion of the gonotrophic cycle [5, 51]. Although our 3D model and structure prediction analysis confer iron and anion binding site/pocket at N-terminal of putative *AcTrf1a*. However, due to the lack of vertebrate transferrin receptor homolog in insects/mosquitoes, it remains unresolved how loading and unloading of iron are achieved at midgut and ovary surfaces, respectively [8, 52]. A recent study by Farkas et.al 2018 suggests the involvement of basally derived endosomes in iron import to the target cells is important in the insect *D*. *melanogaster* and proposes that basal endocytosis is followed by vacuole acidification, a process leading to the activation of ferrireductase for iron release and metabolic utilization [53]. Several studies highlight that transferrin plays an antibacterial immune role against microbial infection in insects [54–56]. Constitutive expression in the fat-body and hemolymph, and a significant upregulation upon bacterial challenge, together indicate that the mosquito transferrin *AcTrf1a* may also have an antibacterial role, similar to vertebrate lactotransferrin [57].

Next, we noticed that the *AcTrf1a* transcript is upregulated after 24 hr of blood meal in the fat body, which may likely facilitate to meet the iron supply for ovarian follicle development during the active vitellogenic process. While endogenous expression enrichment of *Actrf1a* in the ovary after 72 hr of blood-feeding is necessary for the egg development in the matured oocytes. Possibly, this is achieved by uptake of iron from hemolymph as well as synthesizing endogenously in the ovary, though further studies are needed to validate these propositions. Kumara et. al. also reported the sequestered uptake of transferrin by developing oocytes in flesh fly [17]. Next, we tested whether exogenous iron supply through oral supplementation of non-heme iron alters the *AcTrf1a* expression in the fat body and midgut of the naïve adult female mosquito. Here, we observed that the expression remains suppressed until an optimal concentration (1 mM) triggers the upregulation of *AcTrf1a* in the fat body of the naïve mosquito. Corroborating with the previous studies these findings suggest that *AcTrf1a* may also have a role as an antioxidant molecule in the blood-feeding insects [22, 58]. We hypothesized that this iron supplementation and natural blood-feeding together may lead to a synergistic effect on the transcript expression modulation, accelerating iron transportation and ultimately enhancing the reproductive potential.

Supporting the above hypothesis, our data highlighted that oral supplementation of non-heme iron not only upregulates the transcriptional level but also augment the oocyte load in the ovary of gravid adult female mosquitoes. Our functional knockdown experiment showed that there is a significant reduction in oocyte load in the developing ovaries. We propose that transferrin play important role in reproductive physiology of adult female mosquitoes of *An*. *culicifacies*.

## Conclusion

In summary, our results are consistent with previous studies in other mosquitoes and insects suggesting transferrin as a multifunctional protein involved in iron transportation, antibacterial immune protein, and vitellogenesis. We speculated that transferrin may have a physiological role in the trade-off of resources between immunity and reproduction. Possibly this mechanism may facilitate optimal storage and transportation of iron from the midgut/fat-body to the ovaries. Here, we propose that hemocyte originated allelic variant of transferrin plays a pivotal role in mosquito fecundity and survival.

## Supporting information

S1 File (DOCX)
