# Supplementary material for: Functional disruption of transferrin expression alters reproductive physiology in Anopheles culicifacies
Source: PLoS One. 2022 Mar 4;17(3):e0264523. doi: 10.1371/journal.pone.0264523 (PMC8896695; doi:10.1371/journal.pone.0264523)

**Supplementary data**

**Functional disruption of Transferrin gene expression alters reproductive physiology**

**in *Anopheles culicifacies***

Jyoti Rani^1,2^, Tanwee Das De^1^, Charu Chauhan^1^, Seena Kumari^1^, Punita Sharma^1^, Sanjay Tevatiya^1^, Soumyananda Chakraborty^1^, Kailash C Pandey^1^, Namita Singh^2^, Rajnikant Dixit^1^*

**S1 Table:** Protein sequence of *AcTrf1a* used for structural modelling. N-terminal domain were highlighted in red colour. Residues responsible for iron and anion binding were marked in green and blue respectively.

> Transferrin, *Anopheles culicifacies*

AAGVFGGPAIDRQDVQDAAKFRWCVPEELTRICVRLARTAGVPIGCVGGIDRMDCLRKVQNREADYLVADPEDVYVASHFDNQDYIVFSELRTAEEPTAMFR**Y**EGIMLVRASDKFHSLADLRGKRSCH**T**GFG**RNVG**YKIPVTRLQRAGVLKLPADDGSLSPVERELAGLSELFSASCLPGSYSSDAGIDRLLKGRYANLCERCDRPQQCAKDDRYAG**Y**EGAIRCLVENGGDVAFSKTINVRKYFGLPVTPGGLPAGPAVNPNARTEDYLYLCEDGTTRPIADGHPVCSWAQRPWQVLLGNGDLAGAGLRELQTLSQQLRRYWTESNNQISETDRRSAQKLWMDKNAPIVDREKTVAPRDYLNQANYAEVIEREGRFVNVLRLCVVSEDERQKCELMRQAAYSRDIRPALQCVLKTVDACVDAVRDGTQADVVVLRQSNVQLKPLMWETYDDVMVAIADKTITRERLQSGFVALDLSNGQAVAAARLLYSKLPSLTSVDINSSVAAYAPLRIVRSRTLTENSDNAEKVLVCVDLSFQPIANSANCNLESNLNAERNAASVNVRKDVDDALRDSVVHAFTALSEAFGRGQPKEQVFRMFGPFRLRNGEVKQHLIFHDYASELTANK

**S2 Table:** Different physico-chemical properties of *AcTrf1a.*

| **Protein name** | **Total residues** | **pI** | **Mw** | **Net Charge** | **Signal peptide** | **Solubility** | **No. of cystine**  **residues** |
| --- | --- | --- | --- | --- | --- | --- | --- |
| *Trf1a* | 624 | 6.29 | 69261.39 | -3 | - | Soluble* | 18 |

Based on protparam (https://web.expasy.org/protparam/)

**S3 Table:** List of primer sequences used in the study.

| Sr. No. | Target name | Sequence |
| --- | --- | --- |
| 1. | *Trf1a* | Fw- GGTACTCGTTTGTGTGGATT |
|  |  | Rev- AACATACGAAACACCTGCTC |
| 2. | Dsr_*Trf1a* | Fw- TAATACGACTCACTATAGGGGCTATAAGATTCCGGTAACA |
|  |  | Rev- TAATACGACTCACTATAGGGCTCATAACCAGCATATCGAT |
| 3. | Actin | Fw- TGCGTGACATCAAGGAGAAG |
|  |  | Rev- GATTCCATACCCAGGAACGA |

**S4 Table:** Blast result of deduced amino acid sequence of our target *AcTrf1a* against vector base data. Amino acid substitutions were highlights by bold letters and enclosed in box. Yellow and grey highlighted text represent the putative N-glycosylation sites at -Asn- residue present at 493 and 500 position of amino acid.

Trf1a 1 AAGVFGGPAIDRQDVQDAAKFRWCVPEELTRICVRLARTAGVPIGCVGGIDRMDCLRKVQ 60

ACUA023913 13 AAGVFGGPAIDRQDVQDAAKFRWCVPEELTRICVRLARTAGVPIGCVGGIDRMDCLRKVQ 72

Trf1a 61 NREADYLVADPEDVYVASHFDNQDYIVFSELRTAEEPTAMFRYEGIMLVRASDKFHSLAD 120

ACUA023913 73 NREADYLVADPEDVYVASHFDNQDYIVFSELRTAEEPTAMFRYEGIMLVRASDKFHSLAD 132

Trf1a 121 LRGKRSCHTGFGRNVGYKIPVTRLQRAGVLKLPADDGSLSPVERELAGLSELFSASCLPG 180

ACUA023913 133 LRGKRSCHTGFGRNVGYKIPVTRLQRAGVLKLPADDGSLSPVERELAGLSELFSASCLPG 192

Trf1a 181 SYSSDAGIDRLLKGRYANLCERCDRPQQCAKDDRYAGYEGAIRCLVENGGDVAFSKTINV 240

ACUA023913 193 SYSSDAGIDRLLKGRYANLCERCDRPQQCAKDDRYAGYEGAIRCLVENGGDVAFSKTINV 252

Trf1a 241 RKYFGLPVTPGGLPAGPAVNPNARTEDYLYLCEDGTTRPIADGHPVCSWAQRPWQVLLGN 300

ACUA023913 253 RKYFGLPVTPGGLPAGPAVNPNARTEDYLYLCEDGTTRPIADGHPVCSWAQRPWQVLLGN 312

Trf1a 301 GDLAGAGLRELQTLSQQLRRYWTESNNQISETDRRSAQKLWMDKNAPIVDREKTVAPRDY 360

ACUA023913 313 GDLAGAGLRELQTLSQQLRRYWTESNNQISETDRRSAQKLWMDKNAPIVDREKTVAPRDY 372

Trf1a 361 LNQANYAEVIEREGRFVNVLRLCVVSEDERQKCELMRQAAYSRDIRPALQCVLKTVDACV 420

ACUA023913 373 LNQANYAEVIEREGRFVNVLRLCVVSEDERQKCELMRQAAYSRDIRPALQCVLKTVDACV 432

Trf1a 421 DAVRDGT**E**ADVVVLRQSNVQLKPLMWETYDDVMVAIADKTITRERLQSGFVALDLSNGQA 480

ACUA023913 433 DAVRDGT**Q**ADVVVLRQSNVQLKPLMWETYDDVMVAIADKTITRERLQSGFVALDLSNGQA 492

Trf1a 481 VAAARLLYSK**I**P**NLTS**VDI**NSSV**AAYAPLRIVRSRTLTEN**A**DNAEKVLVCVDLSFQPIAN 540

ACUA023913 493 VAAARLLYSK**L**P**SLTS**VDI**NSSV**AAYAPLRIVRSRTLTEN**S**DNAEKVLVCVDLSFQPIAN 552

Trf1a 541 SANCNLESNLNAERNAASVNVRKDVDDALRDSVVHAFTALSEAFGRGQPKEQVFRMFGPF 600

ACUA023913 553 SANCNLESNLNAERNAASVNVRKDVDDALRDSVVHAFTALSEAFGRGQPKEQVFRMFGPF 612

Trf1a 601 RLRNGEVKQHLIFHDYASELTANK 624

ACUA023913 613 RLRNGEVKQHLIFHDYASELTANK 636

**S5 Table:** Iron and anion (CO_3_^-2^) binding residues of *AcTrf1a* compared to *Manduca sexta* *Trf1* (P22297). Insect iron binding site is found different compared to human transferrin iron binding site (P02787). In most of the insects transferrin iron and anion binding sites are clustered in N-terminal domain only*.*

**N lobe C lobe**

**Iron binding** **Anion binding** **Iron binding** **Anion binding**

Human serum transferrin

**D63 Y95 Y188 H249** **T120 R124** **392D 426Y 517Y 585H** **T452 R456**

**Iron binding** **Anion binding**

*Manduca sexta* Trf1

**Y90 Y204 T116 R120 N121 V122 G123**

*Anopheles culicifacies* Trf1a

**Y103 Y218 T129 R133 N134 V135 G136**

**S6 Table:** Table showing percentage identity matrix of selected insects transferrin1 homologs. **(**Percentage identity table was created using ClustalW2-Phylogeny (version 2.1: http://www.ebi.ac.uk/Tools/phylogeny/clustalw2_phylogeny). Md, *Musca domestica*; Gm, *Glosina morsitans*; Bd, *Bactrocera dorsalis*; Cc, *Ceratitis capitata*; Dm, *Drosophila melanogaster*; Aa, *Aedes aegypti*; Cq, *Culex quinquefasciatus*; *AcTrf1a*, *Anopheles culicifacies Trf1a*; ACUA023913, full length TRF1 seq from vector base; As, *Anopheles stephensi*; Ag*, Anopheles gambiae*.

**
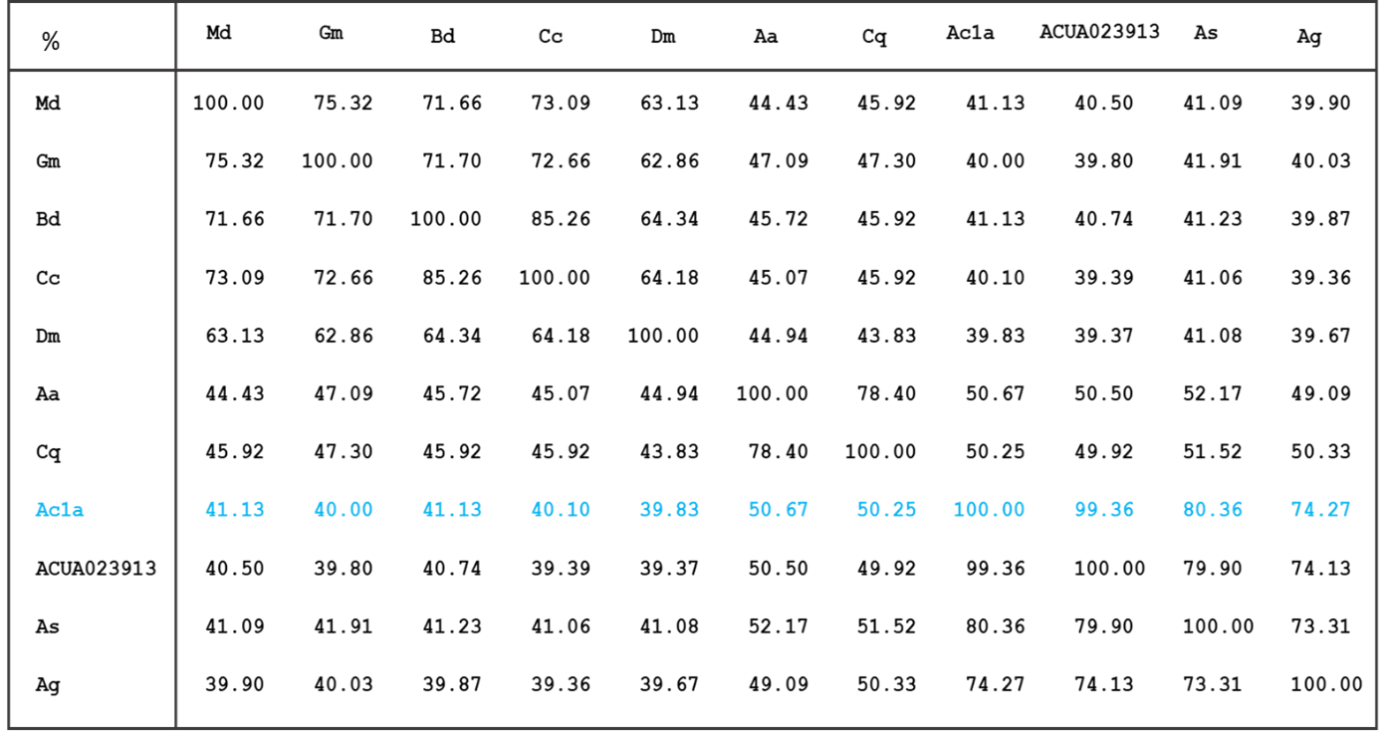
**

**S1 Fig.:** Phylogenetic analysis of both putative transferrin transcripts retrieved from RNA-Seq data of *An. culicifacies* hemocytes. Selected transcripts are highlighted with red circle dot at branch end. *AcTrf1a* show close phylogeny with insects’ homolog while second contig (Ac_Hc_contig_3774) with transferrin fish homologs.


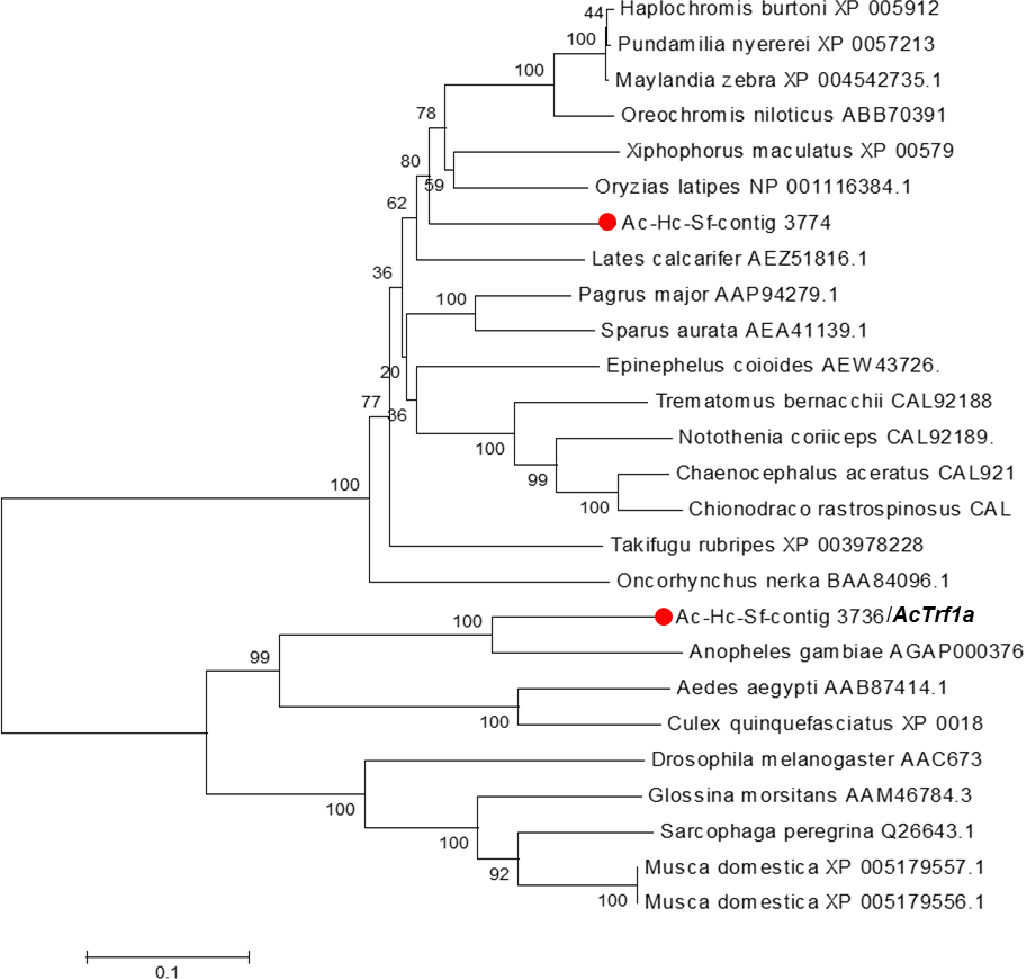


**S2 Fig.:** RT-PCR based expression profiling of *AcTrf1a* in fat body tissue in response to external non heme iron supplementation at lower concentration. Mosquitoes were fed on different concentation of FeCl3 (1µM, 5µM, 10µM) in 10% sugar solution, till the completion of experiment. At 2-3 day of feeding fat body tissue was collected from 20-25 mosquito for relative expression of *AcTrf1a.* Below picture shows that *AcTrf1a* remains unresponsive to this mild FeCl3 concentation and results are insignificant.


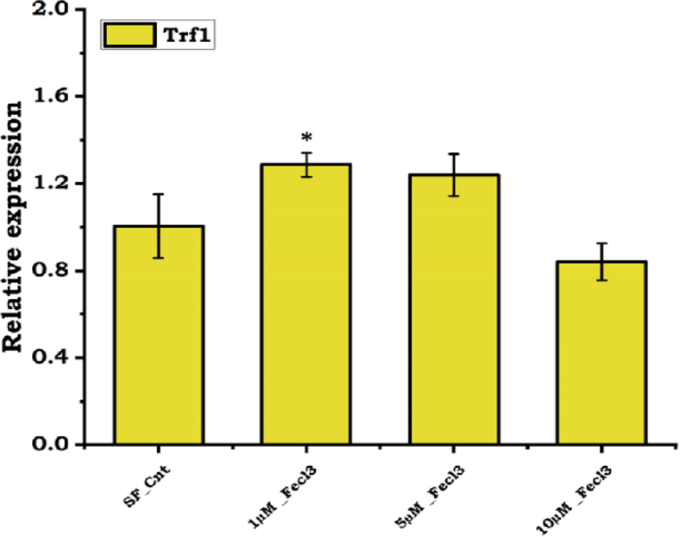


**S3 Fig.:** Phase contrast microscope-based analysis of follicle/oocyte development post AcTrf1a silencing compared to control. Mosquitoes were injected with 2-3 µg of *dsTrf1a* using Nano-injector facility for silencing the target gene compared to *dsLacZ* control. At 3-4 days post silencing mosquitoes of both test and control group was allowed to feed on rabbit host for blood meal uptake and subsequently ovary development analysis was done. Ovaries of 10 mosquitoes from both groups were dissected in 1x PBS under dissecting microscope and stained with 0.5% neutral red for 10 sec. Afterward, stained ovaries were washed with PBS and pictures were taken using Smart phone cameras through the eyepiece of the microscope. (a &b) represents the 10X image of stained intact ovaries without coverslip while; (c) depicts the 10x image of dissected ovaries covered with coverslip; (d) shows 4x image of ovaries without coverslip. Altogether, indicates that there is significant reduction in oocyte post *AcTrf1a* silencing compared to control ovaries.


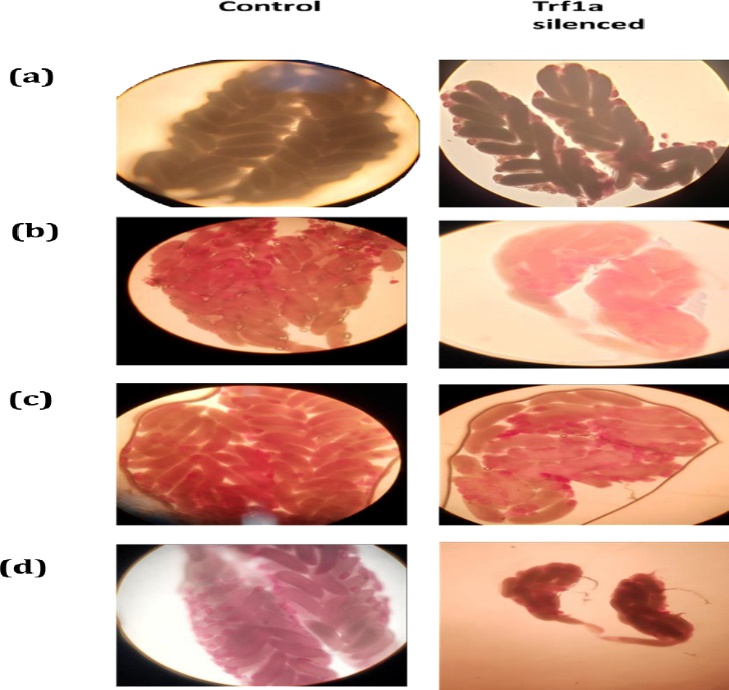


**S4 Fig.:** RT-PCR based expression profiling of *AcTrf1a* in midgut tissue in response to external iron supplement (n=30-35, N2). Post supplementation sample was collected. Above picture shows that *AcTrf1a* remains unresponsive to external iron supplemntation. (*n*=represents the number of mosquitoes pooled for sample collection; *N*= number of replicates).


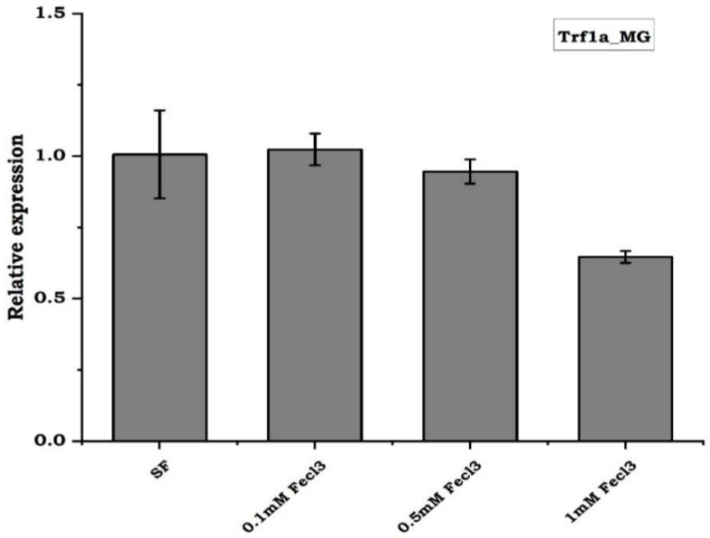


**S5 Fig.:** (a) Real time-PCR based validation of *AcTrf1a* in fat-body tissue after external iron supplementation, *dsrAcTrf1a* injection, and dsr injection followed by iron supplementation to newly emerged mosquitoes in comparison to same age group *dsrLacZ* injected (n=25, N=3); (b) Dot plot showing the comparative oocyte count enhancement in iron supplemented diet and reduction post *AcTrf1a* knockdown in female ovary tissue compared to blood-fed control mosquito group. Post dsRNA injection mosquitoes were fed on blood meal and ovary assessment was done, (n=10, *N*=2). Data represented in the figure were from three independent biological replicates, where each dot is equivalent to replicate. Statistical analysis was done using one-way ANOVA, multiple comparison has been done. *viz* ∗p < 0.05; ∗∗p < 0.005; and ∗∗∗p < 0.0005, (*n*=represents the number of mosquitoes pooled for sample collection; *N*= number of replicate.


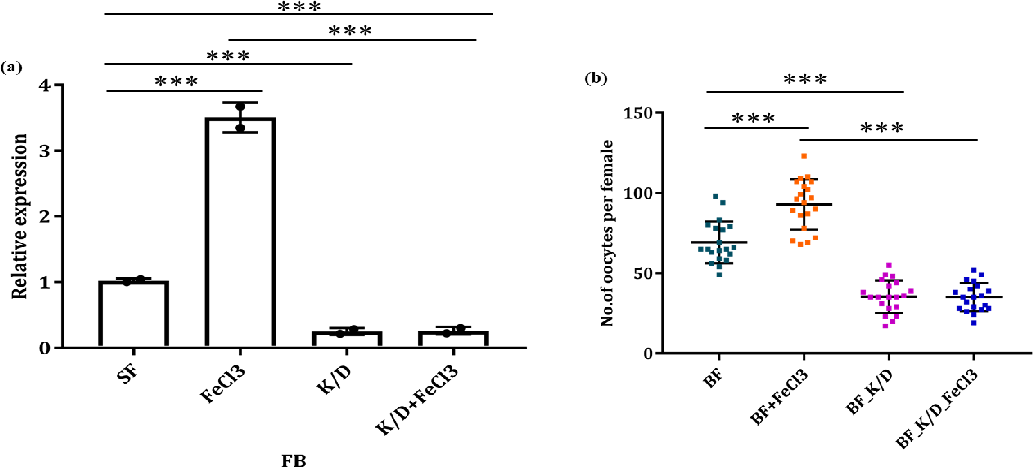


**S6 Fig.:** Multiple sequence alignment of full length *AcTrf1a* and other transferrin homolog sequences selected from mosquitoes and non-mosquito insects species. Full length alignment showed presence of conserved region.


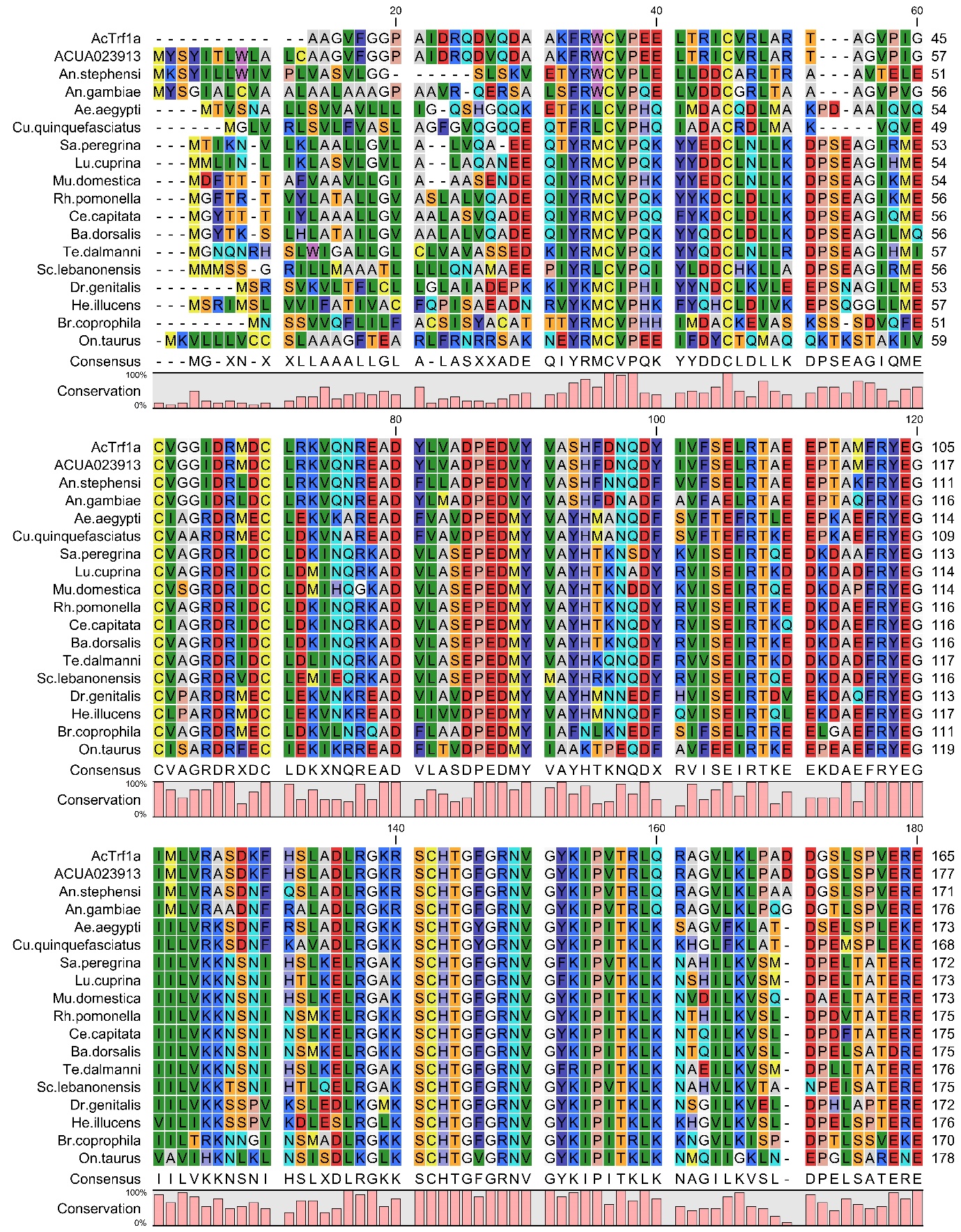


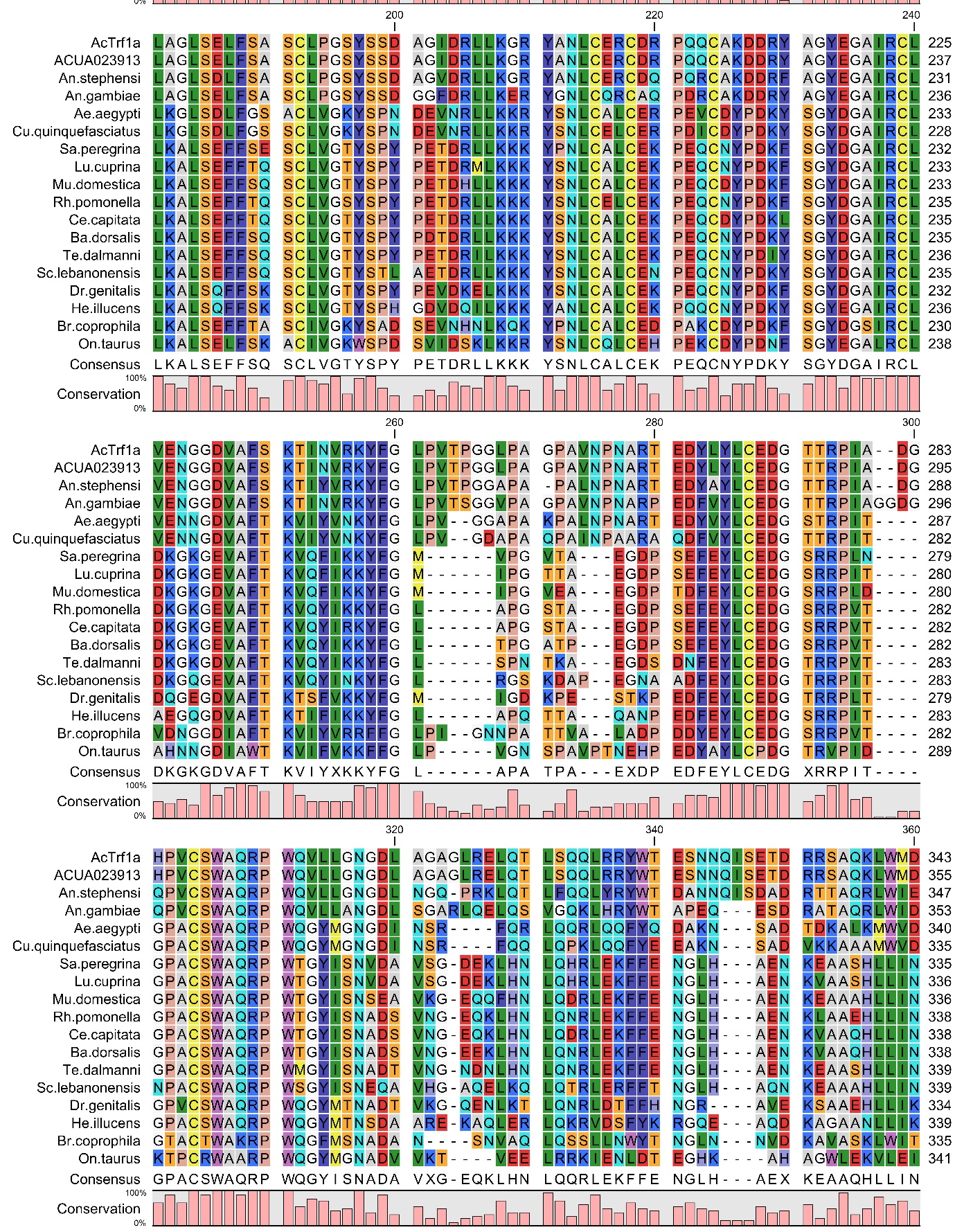


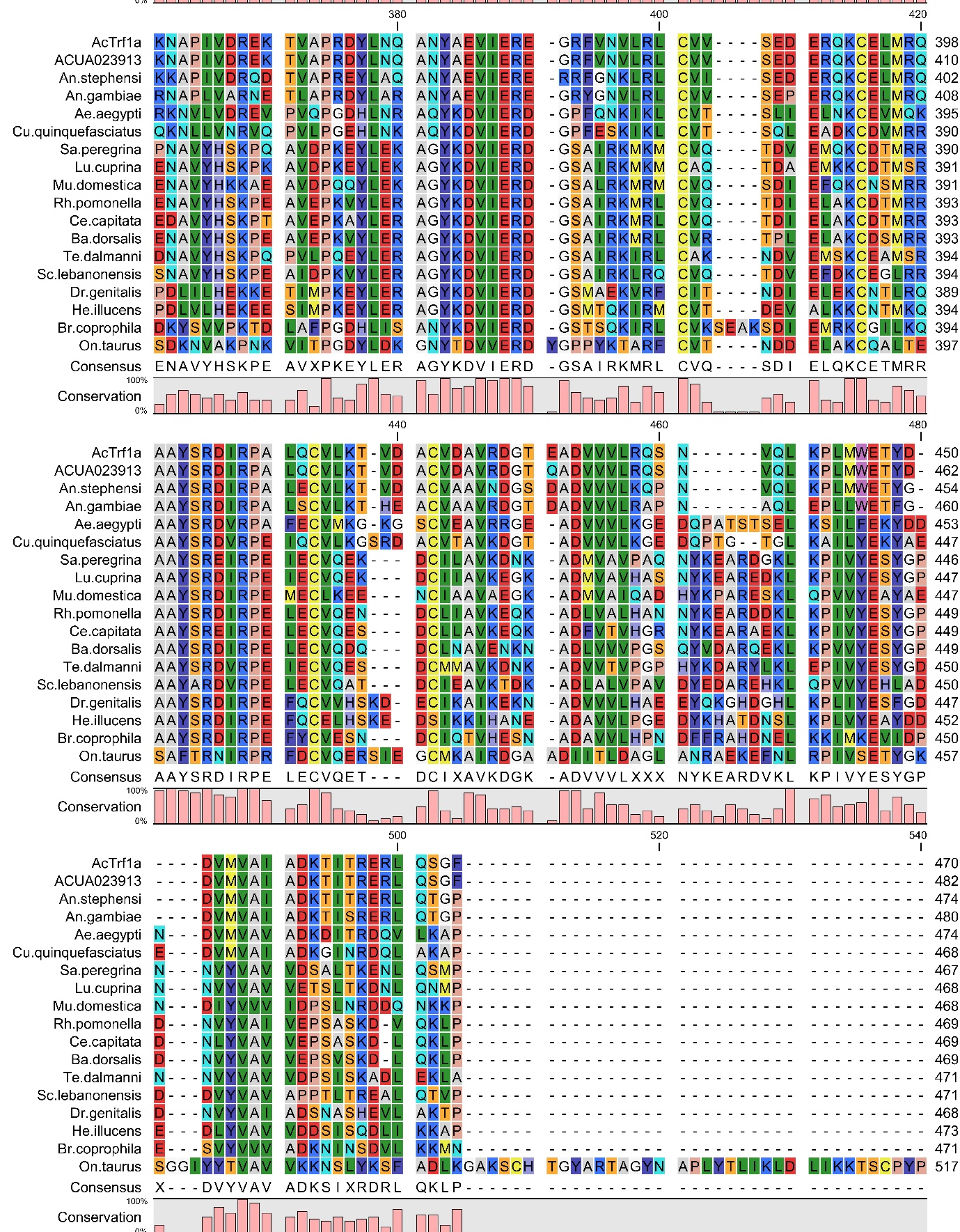


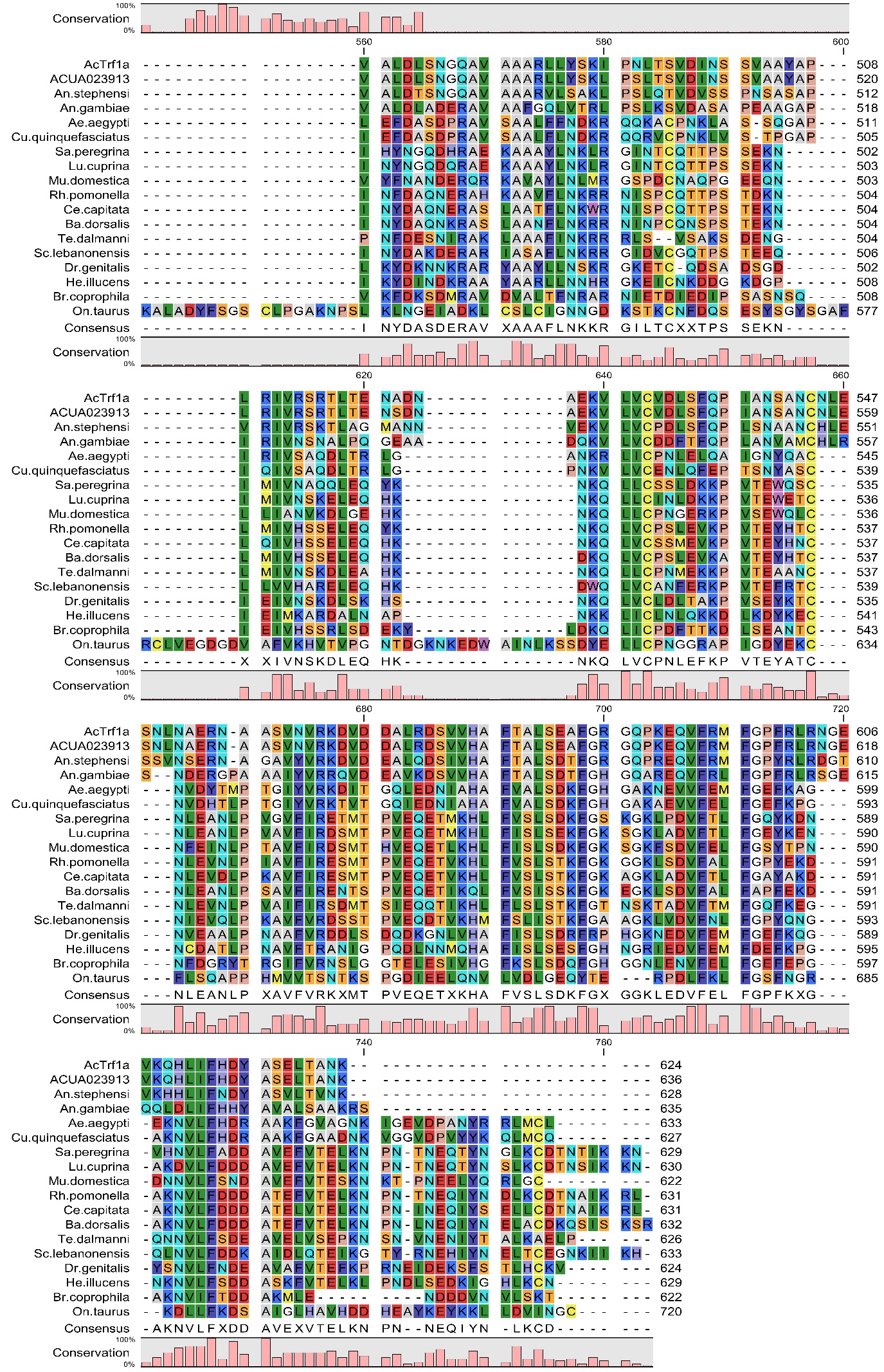

Supplement: S1 File — (DOCX) [file pone.0264523.s001.docx]
